# Ramucirumab after prior sorafenib in patients with advanced hepatocellular carcinoma and elevated alpha-fetoprotein: Japanese subgroup analysis of the REACH-2 trial

**DOI:** 10.1007/s00535-020-01668-w

**Published:** 2020-02-27

**Authors:** Masatoshi Kudo, Takuji Okusaka, Kenta Motomura, Izumi Ohno, Manabu Morimoto, Satoru Seo, Yoshiyuki Wada, Shinpei Sato, Tatsuya Yamashita, Masayuki Furukawa, Takeshi Aramaki, Seijin Nadano, Kazuyoshi Ohkawa, Hirofumi Fujii, Toshihiro Kudo, Junji Furuse, Hiroki Takai, Gosuke Homma, Reigetsu Yoshikawa, Andrew X. Zhu

**Affiliations:** 1 Department of Gastroenterology and Hepatology, Kindai University Faculty of Medicine, 377-2 Ohno-Higashi, Osaka-Sayama, Osaka 589-8511 Japan; 2 National Cancer Center Hospital, Tokyo, Japan; 3 Aso Iizuka Hospital, Fukuoka, Japan; 4 National Cancer Center Hospital East, Chiba, Japan; 5 Kanagawa Cancer Center, Yokohama, Japan; 6 Graduate School of Medicine, Kyoto University, Kyoto, Japan; 7 National Hospital Organization Kyushu Medical Center, Fukuoka, Japan; 8 Kyoundo Hospital, Sasaki Institute, Tokyo, Japan; 9 Graduate School of Medicine, Kanazawa University, Kanazawa, Japan; 10 National Hospital Organization Kyushu Cancer Center, Fukuoka, Japan; 11 Shizuoka Cancer Center Hospital, Shizuoka, Japan; 12 National Hospital Organization Shikoku Cancer Center, Matsuyama, Japan; 13 Osaka International Cancer Institute, Osaka, Japan; 14 Jichi Medical University, Tochigi, Japan; 15 Graduate School of Medicine, Osaka University, Osaka, Japan; 16 Kyorin University Faculty of Medicine, Tokyo, Japan; 17 Eli Lilly Japan K.K., Kobe, Japan; 18 Massachusetts General Hospital Cancer Center, Harvard Medical School, Boston, MA USA

**Keywords:** Alpha-fetoprotein, Hepatocellular carcinoma, Japanese subanalysis, Ramucirumab, VEGFR2

## Abstract

**Background:**

The global, randomized, phase 3 REACH-2 study (ClinicalTrials.gov identifier: NCT02435433) found significantly longer overall survival (OS) for second-line ramucirumab versus placebo (hazard ratio [HR]: 0.710, 95% confidence interval [CI] 0.531–0.949, *P* = 0.0199) in patients with advanced hepatocellular carcinoma (HCC) and alpha-fetoprotein (AFP) ≥ 400 ng/mL. This prespecified subgroup analysis evaluated the efficacy and safety of ramucirumab in the Japanese patients enrolled in the study.

**Methods:**

Patients with advanced HCC and AFP ≥ 400 ng/mL after first-line sorafenib were randomized 2:1 to ramucirumab (8 mg/kg intravenously) or placebo every 2 weeks. Hazard ratios for progression-free survival (PFS) and OS (primary endpoint of the overall study) were estimated using the stratified Cox regression model. We also pooled individual patient data from REACH-2 with data from REACH (NCT01140347) for patients with AFP ≥ 400 ng/mL.

**Results:**

In the Japanese REACH-2 subpopulation, there were improvements for ramucirumab (*n* = 41) versus placebo (*n* = 18) in PFS (HR 0.282, 95% CI 0.144–0.553) and OS was numerically prolonged (HR 0.599, 95% CI 0.303–1.187), consistent with the significant benefit seen in the overall REACH-2 study population. In the ramucirumab and placebo arms, respectively, the objective response rate was 7.3% and 0%, and the disease control rate was 70.7% and 33.3%. The most frequently reported grade ≥ 3 treatment-emergent adverse event was hypertension (ramucirumab: 15%; placebo: 11%).

**Conclusions:**

Ramucirumab after prior sorafenib improved PFS and OS compared with placebo, with a manageable safety profile, in the Japanese REACH-2 subpopulation, consistent with the overall REACH-2 study results. Ramucirumab is the first agent to demonstrate clinical benefit for Japanese patients with HCC in the second-line setting.

**Electronic supplementary material:**

The online version of this article (10.1007/s00535-020-01668-w) contains supplementary material, which is available to authorized users.

## Introduction

Conventional chemotherapy is of limited or no benefit in patients with advanced hepatocellular carcinoma (HCC) [1]. For a long time, sorafenib, a multikinase inhibitor, was the only systemic treatment shown to improve overall survival (OS) in patients with HCC [1-3]. However, sorafenib is associated with significant toxicity, with approximately 30% of patients stopping treatment because of intolerance [4]. Since 2017, new agents for HCC treatment have emerged, with the approval (including in Japan) of the multikinase inhibitors lenvatinib and regorafenib in the first- and second-line settings, respectively, and the investigation of immuno-oncology agents as monotherapy and in combination with molecular targeted agents [5]. Most recently, cabozantinib was approved for patients with HCC who have previously received sorafenib in the United States in January 2019, based on the CELESTIAL trial [6, 7]. As the CELESTIAL trial did not include Japanese patients, cabozantinib is not yet approved in Japan; clinical trials assessing its efficacy and safety in Japanese patients are ongoing [8]. Similar to sorafenib, lenvatinib, and regorafenib are associated with significant toxicities, with all three multikinase inhibitors associated with hypertension, hand-foot skin reactions, diarrhea, fatigue, and decreased appetite/anorexia [2, 9-11]. Japanese patients with advanced HCC tend to be older and more likely to discontinue multikinase inhibitor treatment early owing to toxicity (e.g., hand-foot skin reactions, diarrhea) than patients from other countries [12-14]. Therefore, new treatment options that are effective and well-tolerated are needed for Japanese patients with HCC.

Vascular endothelial growth factor (VEGF)- and VEGF receptor 2 (VEGFR2)–mediated angiogenesis contribute to the pathogenesis of HCC [15], making this signaling pathway a possible therapeutic target. Elevated alpha-fetoprotein (AFP) is associated with increased VEGF and VEGFR2 signaling, and poor prognosis, in HCC [16, 17]. Whereas the most common approach to inhibiting the VEGF-VEGFR2 signaling pathway in HCC is inhibition of VEGFR-2 tyrosine kinases and their downstream targets by small molecule/multitargeted tyrosine kinase receptor inhibitors, ramucirumab (a human IgG1 monoclonal antibody) inhibits ligand activation of VEGFR2 [18]. In the global, randomized, phase 3 REACH study (*N* = 565) conducted in patients with advanced HCC previously treated with sorafenib, ramucirumab did not significantly improve OS compared with placebo (hazard ratio [HR] 0.87, 95% confidence interval [CI] 0.72–1.05, *P* = 0.14) [19]. However, a prespecified subgroup analysis showed a numerical improvement in OS for ramucirumab versus placebo in the subgroup of patients with baseline AFP ≥ 400 ng/mL (*n* = 250; HR 0.67, 95% CI 0.51–0.90) [19]. Based on this result, the global, randomized, phase 3 REACH-2 study (*N* = 292) was conducted in patients with HCC previously treated with sorafenib who had a baseline AFP level ≥ 400 ng/mL [20]. Ramucirumab significantly improved OS compared with placebo in the overall REACH-2 study population (HR 0.710, 95% CI 0.531–0.949, *P* = 0.0199) [20], confirming the survival benefit for ramucirumab observed in the subgroup of patients in the REACH study with AFP ≥ 400 ng/mL [19].

To evaluate the efficacy and safety of ramucirumab in Japanese patients with HCC, a prespecified subgroup analysis of the Japanese patients enrolled in the REACH-2 study was conducted. In addition, to evaluate the efficacy and safety of ramucirumab in a larger population of Japanese patients, the efficacy and safety results for the Japanese REACH-2 subpopulation and the Japanese REACH subpopulation with baseline AFP ≥ 400 ng/mL were pooled; these post hoc analyses are also presented.

## Methods

### Study design

Full details of the REACH-2 and REACH study design have been published elsewhere [19, 20]. REACH-2 is a randomized, double-blind, placebo-controlled, phase 3 study of second-line ramucirumab treatment in patients with HCC and elevated baseline AFP following first-line treatment with sorafenib, conducted in 20 countries. The ethics review board of each site approved the study protocol. The study was conducted in accordance with the Declaration of Helsinki, the Council for International Organizations of Medical Sciences International Ethical Guidelines, Good Clinical Practice guidelines, and applicable local guidelines. All patients provided written informed consent before enrollment. The study was registered at www.clinicaltrials.gov (NCT02435433).

## Study population

Briefly, patients were adults (≥ 18 years of age) with histopathologically or cytologically confirmed HCC (or a diagnosis of cirrhosis and HCC with classical imaging characteristics), Barcelona Clinic Liver Cancer stage B or C disease, Child–Pugh class A liver disease, Eastern Cooperative Oncology Group performance status of 0 or 1, and elevated AFP (≥ 400 ng/mL). Patients had received prior sorafenib therapy (≥ 14 days) that was discontinued due to disease progression or intolerance.

## Randomization and procedures

Patients were randomized in a 2:1 ratio to receive ramucirumab or placebo. Randomization was stratified by region (Region 1: the Americas, Europe, Israel, and Australia; Region 2: Asia except Japan; and Region 3: Japan), Eastern Cooperative Oncology Group performance status (0 vs. 1), and macrovascular invasion (yes vs. no). Patients, investigators, and the sponsor study team were blinded to individual treatment assignments for the study duration. Patients received intravenous ramucirumab 8 mg/kg or placebo over approximately 60 min every 2 weeks plus best supportive care. Treatment continued until disease progression, unacceptable toxicity, noncompliance, patient withdrawal of consent, or other protocol-specified discontinuation criteria were met. Tumor response was assessed by computed tomography or magnetic resonance imaging every 6 weeks during the first 6 months of treatment and every 9 weeks thereafter, according to Response Evaluation Criteria in Solid Tumors (RECIST) version 1.1.

### Statistical analysis

Prespecified efficacy assessments were conducted on the Japanese intent-to-treat population, comprising all randomized patients enrolled in Japan. Safety assessments were conducted on the Japanese safety population, comprising all randomized patients enrolled in Japan who received ≥ 1 dose of study drug. Survival curves and median with 95% CIs were estimated for progression-free survival (PFS), OS, and time-to-progression (TTP) using the Kaplan–Meier method. The PFS, OS, and TTP HRs were estimated using the stratified Cox regression model. The OS HR was also estimated after adjusting for baseline AFP levels. The objective response rate (ORR) was the percentage of patients with a best overall response of complete response or partial response; the disease control rate (DCR) was the percentage of patients with a best overall response of complete response, partial response, or stable disease. Adverse events were summarized as the number and percentage of patients reporting each event. Post hoc pooled analyses of the efficacy and safety results for the Japanese REACH-2 subpopulation and the Japanese REACH subpopulation with AFP ≥ 400 ng/mL (ClinicalTrials.gov identifier: NCT01140347) were conducted at the individual patient data level. Analyses were conducted using SAS Version 9.4 (SAS Institute, Cary, NC, USA).

## Results

### Demographic and baseline clinical characteristics

A total of 59 Japanese patients were enrolled in the REACH-2 study, 41 patients in the ramucirumab arm and 18 patients in the placebo arm (Table 1). All randomized Japanese patients received study treatment and thus constituted both the intent-to-treat and safety populations. Baseline characteristics were generally similar between the two arms of the Japanese REACH-2 subpopulation; however, the proportion of male patients was lower, the proportion of patients with Child–Pugh score A-5 was higher, the median duration of disease was longer, the percentage of patients discontinuing sorafenib treatment because of progressive disease was higher, and the median baseline AFP level was higher in the ramucirumab arm than in the placebo arm (Table 1).

**Table 1:** Baseline characteristics of the Japanese REACH-2 subpopulation

Characteristic^a^	Ramucirumab (*n* = 41)	Placebo (*n* = 18)
Sex		
Male	31 (76)	16 (89)
Female	10 (24)	2 (11)
Age, years		
Median	71	68
Minimum–maximum	(39–84)	(40–82)
ECOG PS		
0	33 (80)	15 (83)
1	8 (20)	3 (17)
Child–Pugh score		
A-5	28 (68)	7 (39)
A-6	13 (32)	11 (61)
BCLC stage		
Stage B	9 (22)	4 (22)
Stage C	32 (78)	14 (78)
Etiology^b^		
Hepatitis B	13 (32)	6 (33)
Hepatitis C	15 (37)	8 (44)
Significant alcohol use	4 (10)	3 (17)
Steatohepatitis (NASH, fatty liver)	4 (10)	0
Primary biliary cirrhosis	1 (2)	0
Hepatitis, non-A, non-B, non-C	2 (5)	1 (6)
Cryptogenic cirrhosis	1 (2)	1 (6)
Others	5 (12)	1 (6)
Macrovascular invasion present		
Yes	9 (22)	3 (17)
No	32 (78)	15 (83)
Extrahepatic spread present		
Yes	27 (66)	13 (72)
No	14 (34)	5 (28)
Duration of disease (months)		
Median	43.70	26.76
Minimum–maximum	2.27–180.17	3.35–160.10
Reason for discontinuation of sorafenib		
Progressive disease	36 (88)	13 (72)
Intolerance	5 (12)	5 (28)
Duration of prior sorafenib treatment (months)		
Median	5.29	5.32
Minimum–maximum	1.08–44.42	0.49–25.43
AFP (ng/mL)		
Median	7942	2483
Minimum–maximum	436–230,500	489–246,100

*AFP* alpha-fetoprotein, *BCLC* Barcelona Clinic Liver Cancer, *ECOG* Eastern Cooperative Oncology Group, *NASH* nonalcoholic steatohepatitis, *PS* performance status

^a^Except where otherwise indicated, data are *n* (%)

^b^The sum of the percentages was more than 100% because patients could report more than one etiology

## Efficacy

**Fig. 1:**
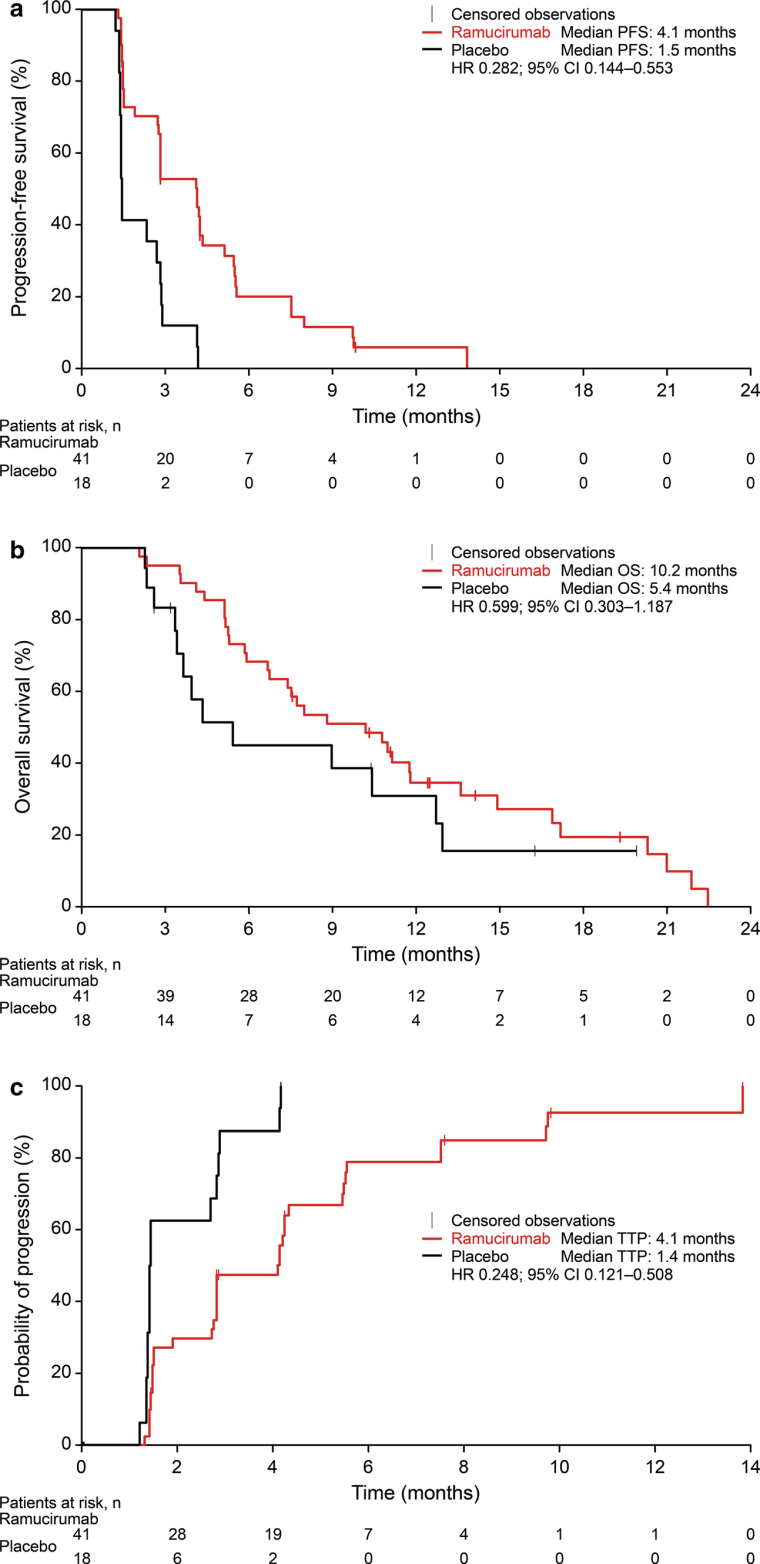
Kaplan–Meier plots of progression-free survival (**a**), overall survival (**b**), and time-to-progression (**c**) in Japanese patients receiving ramucirumab or placebo in the REACH-2 study. *CI* confidence interval, *HR* hazard ratio, *OS* overall survival, *PFS* progression-free survival, *TTP* time-to-progression

In the Japanese REACH-2 subpopulation, PFS and TTP were longer, and OS was numerically improved, in the ramucirumab arm compared with the placebo arm (Fig. 1). Median PFS was 4.1 versus 1.5 months for ramucirumab versus placebo (HR 0.282, 95% CI 0.144–0.553) (Fig. 1a). Median OS was 10.2 versus 5.4 months for ramucirumab versus placebo (HR 0.599, 95% CI 0.303–1.187) (Fig. 1b). Analysis adjusting for the baseline AFP level supported the clinically meaningful survival benefit observed for ramucirumab in the Japanese REACH-2 subpopulation. The adjusted OS HR was 0.531 (95% CI 0.266–1.057), with median OS of 10.4 versus 6.7 months for ramucirumab versus placebo. Median TTP was 4.1 versus 1.4 months for ramucirumab versus placebo (HR 0.248, 95% CI 0.121–0.508) (Fig. 1c). In total, 3 of 41 patients in the ramucirumab arm responded for an ORR of 7.3% (95% CI 0–15.3). No patients responded in the placebo arm. The DCR was higher in the ramucirumab arm (70.7%, 95% CI 56.8–84.7) than the placebo arm (33.3%, 95% CI 11.6–55.1).

## Treatment exposure

In the Japanese REACH-2 subpopulation, the median number (interquartile range) of treatment cycles was higher in the ramucirumab arm (7 cycles [3-13]) than in the placebo arm (3 cycles [3, 4]). Median relative dose intensity was high in both arms (ramucirumab: 98.4%: placebo: 100.3%).

### Prior therapy before sorafenib treatment and postdiscontinuation therapy

In the Japanese REACH-2 subpopulation, 81% of patients received locoregional therapy before sorafenib treatment, which included 71% of patients receiving transarterial chemoembolization (TACE).

In the Japanese REACH-2 subpopulation, 10 of 41 patients (24%) in the ramucirumab arm and 6 of 18 patients (33%) in the placebo arm received post-treatment anticancer systemic therapies (Supplementary Table 1), including chemotherapy (4 of 41 patients, 10% vs. 2 of 18 patients, 11%), immunotherapy (4 of 41 patients, 10% vs. 1 of 18 patients, 6%), and targeted therapy (7 of 41 patients, 17% vs. 4 of 18 patients, 22%).

## Safety

In the Japanese REACH-2 subpopulation, the incidence of treatment-emergent adverse events (TEAEs) of grade ≥ 3 was higher in the ramucirumab arm than in the placebo arm (Table 2). The most frequently reported TEAEs in the ramucirumab arm were decreased appetite, peripheral edema, hypertension, and malaise (Table 2). The incidence of adverse events of special interest (AESIs) was mostly higher in the ramucirumab arm than in the placebo arm, including liver injury/liver failure (largely low-grade ascites), bleeding/hemorrhage events (largely epistaxis), hypertension, and proteinuria (Table 2).

**Table 2:** Summary of adverse events in the Japanese REACH-2 subpopulation

	Ramucirumab (*n* = 41), *n* (%)	Placebo (*n* = 18), *n* (%)
Discontinuations due to treatment-related AE	4 (10)	1 (6)
Deaths due to treatment-related AE	0	0

TEAEs in ≥ 15% patients in the ramucirumab arm	Any grade	Grade ≥ 3	Any grade	Grade ≥ 3
Patients with ≥ 1 TEAE	39 (95)	25 (61)	16 (89)	8 (44)
Decreased appetite	13 (32)	2 (5)	6 (33)	0
Peripheral edema	12 (29)	0	4 (22)	0
Hypertension	11 (27)	6 (15)	2 (11)	2 (11)
Malaise	11 (27)	0	4 (22)	0
Ascites	10 (24)	0	0	0
Constipation	10 (24)	0	5 (28)	0
Epistaxis	9 (22)	0	1 (6)	0
Hypoalbuminemia	9 (22)	0	2 (11)	1 (6)
Diarrhea	8 (20)	0	4 (22)	0
Nausea	8 (20)	0	2 (11)	0
Pyrexia	8 (20)	0	0	0
Insomnia	7 (17)	0	1 (6)	0
Proteinuria	7 (17)	0	1 (6)	0
AESIs^a^				
Liver injury/liver failure	19 (46)	11 (27)	5 (28)	3 (17)
Ascites	10 (24)	0	0	0
Hepatic encephalopathy	3 (7)	3 (7)	0	0
Bleeding/hemorrhage events	14 (34)	2 (5)	4 (22)	1 (6)
Epistaxis	9 (22)	0	1 (6)	0
Hypertension	11 (27)	6 (15)	2 (11)	2 (11)
Proteinuria	7 (17)	0	1 (6)	0
GI hemorrhage events	4 (10)	2 (5)	2 (11)	1 (6)
Arterial thromboembolic events	1 (2)	0	0	0
Infusion-related reactions^b^	1 (2)	0	0	0
Congestive heart failure	1 (2)	1 (2)	0	0
Pulmonary hemorrhage events	1 (2)	0	1 (6)	0
Venous thromboembolic events	1 (2)	0	0	0
Fistula	0	0	0	0
GI perforation	0	0	0	0

*AE* adverse event, *AESI* adverse event of special interest, *GI* gastrointestinal, *MedDRA* Medical Dictionary for Regulatory Activities, *TEAE* treatment-emergent adverse event

^a^AESI consolidated category term or MedDRA preferred term

^b^Infusion-related reactions occurring within 24 h of infusion

### Pooled analyses

**Fig. 2:**
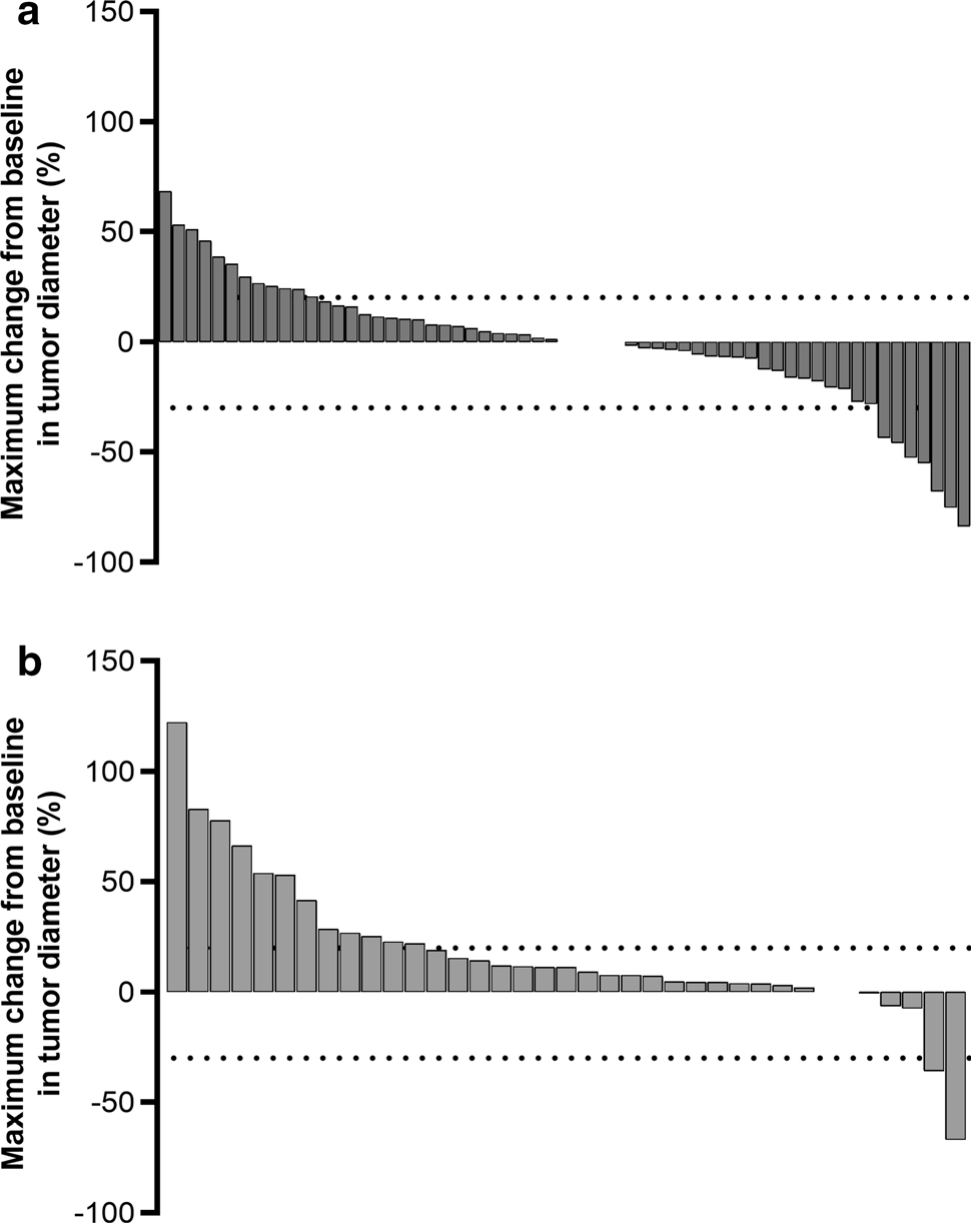
Waterfall plots of maximum change from baseline in Japanese patients receiving ramucirumab (**a**) or placebo (**b**) in the REACH-2 and REACH (baseline AFP ≥ 400 ng/mL) studies. *AFP* alpha-fetoprotein

**Fig. 3:**
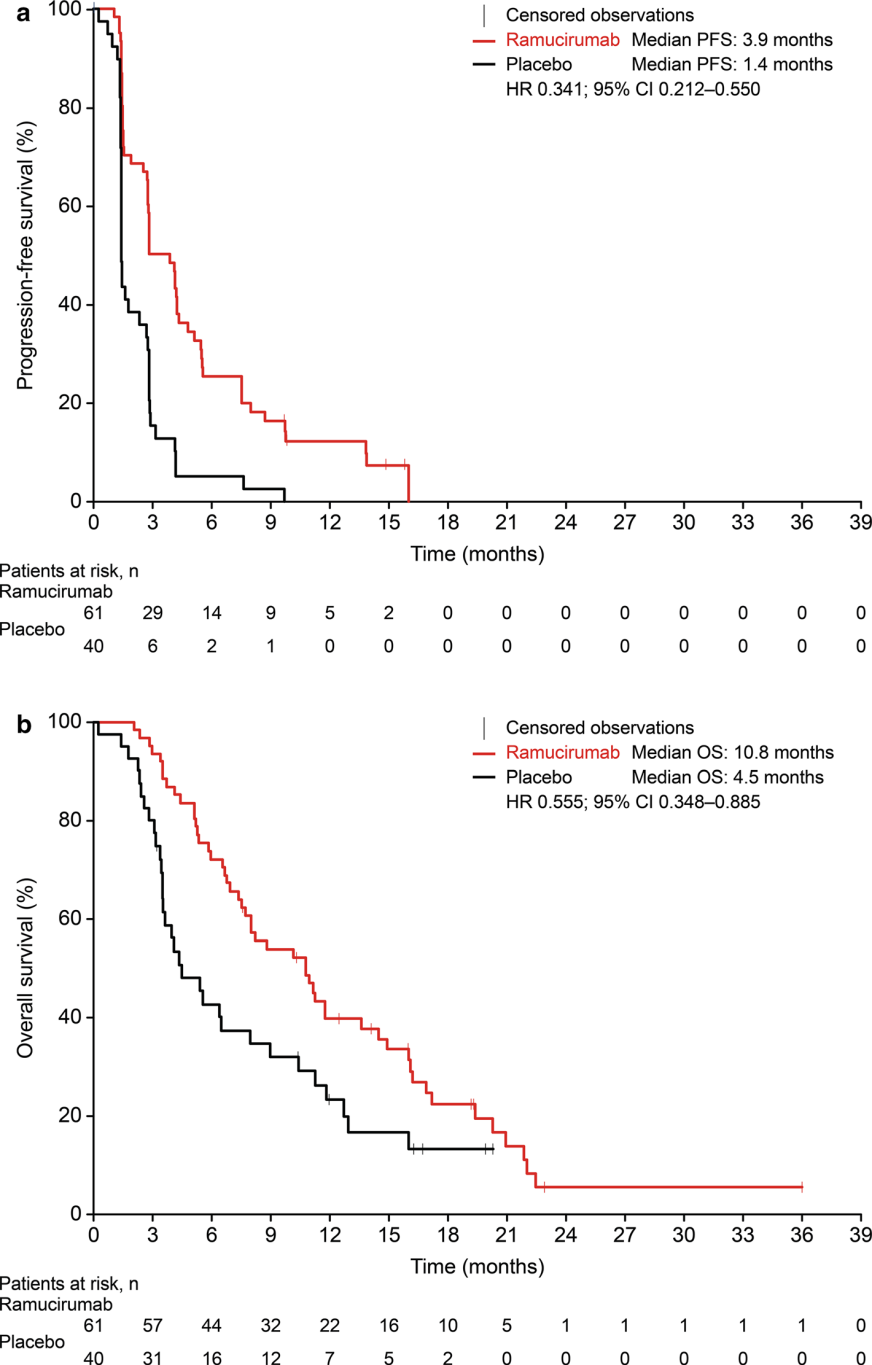
Kaplan–Meier plots of PFS (**a**) and OS (**b**) in Japanese patients receiving ramucirumab or placebo in the REACH-2 and REACH (baseline AFP ≥ 400 ng/mL) studies. *AFP* alpha-fetoprotein, *CI* confidence interval, *HR* hazard ratio, *OS* overall survival, *PFS* progression-free survival

Pooling the individual patient data for the Japanese REACH-2 subpopulation and the Japanese REACH subpopulation with baseline AFP ≥ 400 ng/mL resulted in a combined population with 61 patients in the ramucirumab arm and 40 patients in the placebo arm. The baseline characteristics for the pooled Japanese REACH-2/REACH subpopulations were similar to those for the Japanese REACH-2 subpopulation (Table 3). In pooled efficacy analyses, the ORR was 9.8% versus 2.5% and the DCR was 67.2% versus 35.0% for ramucirumab versus placebo. A greater proportion of patients experienced a reduction in tumor size in the ramucirumab arm (Fig. 2a) compared with the placebo arm (Fig. 2b). PFS and OS were longer in the ramucirumab arm compared with the placebo arm in the pooled Japanese REACH-2/REACH subpopulations (median PFS 3.9 vs. 1.4 months, HR 0.341, 95% CI 0.212–0.550; median OS 10.8 vs. 4.5 months, HR 0.555, 95% CI 0.348–0.885) (Fig. 3).

**Table 3:** Baseline characteristics of the Japanese REACH-2 subpopulation and the Japanese REACH subpopulation with baseline AFP ≥ 400 ng/mL

Characteristic^a^	Ramucirumab (*n* = 61)	Placebo (*n* = 40)^b^
Sex		
Male	45 (74)	35 (88)
Female	16 (26)	5 (13)
Age, years		
Median	69	68
Minimum–maximum	(39–84)	(26–82)
ECOG PS		
0	46 (75)	33 (83)
1	15 (25)	7 (18)
Child–Pugh score		
A-5	35 (57)	20 (50)
A-6	25 (41)	19 (48)
B-7	0 (0)	1 (3)
B-8	1 (2)	0 (0)
BCLC stage		
Stage B	13 (21)	8 (20)
Stage C	48 (79)	32 (80)
Etiology^c^		
Hepatitis B	19 (31)	14 (35)
Hepatitis C	28 (46)	18 (45)
Significant alcohol use	5 (8)	6 (15)
Steatohepatitis (NASH, fatty liver)	4 (7)	1 (3)
Others	9 (15)	5 (13)
Macrovascular invasion present		
Yes	16 (26)	8 (20)
No	45 (74)	32 (80)
Extrahepatic spread present		
Yes	38 (62)	30 (75)
No	23 (38)	10 (25)
Duration of disease (months)		
Median	41.56	22.74
Minimum–maximum	2.27–180.17	0.10–160.10
Reason for discontinuation of sorafenib		
Progressive disease	54 (89)	33 (83)
Intolerance	7 (11)	7 (18)
Duration of prior sorafenib treatment (months)		
Median	3.52	4.99
Minimum–maximum	0.79–44.42	0.49–25.43
AFP (ng/mL)		
Median	7376	3029
Minimum–maximum	413–230,500	489–628,390

*AFP* alpha-fetoprotein, *BCLC* Barcelona Clinic Liver Cancer, *ECOG* Eastern Cooperative Oncology Group, *NASH* nonalcoholic steatohepatitis, *PS* performance status

^a^Except where otherwise indicated, data are *n* (%)

^b^The sum of the percentages may be more than 100% because of rounding

^c^The sum of the percentages was more than 100% because patients could report more than one etiology

In the pooled Japanese REACH-2/REACH subpopulations, the PFS HR was 0.158 (95% CI 0.030–0.825) for patients who were intolerant to sorafenib (ramucirumab: 7 patients; placebo: 7 patients) and 0.385 (95% CI 0.231–0.642) for patients who progressed on sorafenib treatment (ramucirumab: 54 patients: placebo: 33 patients). The OS HR was 0.652 (95% CI 0.190–2.231) for patients who were intolerant to sorafenib and 0.545 (95% CI 0.327–0.910) for patients who progressed on sorafenib treatment.

In the pooled safety analyses, the incidence and severity of the commonly reported TEAEs and AESIs in the ramucirumab arm of the combined Japanese REACH-2/REACH subpopulations (Table 4) were similar to those reported in the ramucirumab arm of the Japanese REACH-2 subpopulation. No new safety signals were identified.

**Table 4:** Summary of adverse events in the Japanese REACH-2 subpopulation and the Japanese REACH subpopulation with baseline AFP ≥ 400 ng/mL

	Ramucirumab (*n* = 61), *n* (%)		Placebo (*n* = 40), *n* (%)	
TEAEs in ≥ 15% patients in the ramucirumab arm	Any grade	Grade ≥ 3	Any grade	Grade ≥ 3
Patients with ≥ 1 TEAE	59 (97)	34 (56)	37 (93)	18 (45)
Ascites	20 (33)	2 (3)	1 (3)	1 (3)
Peripheral edema	20 (33)	0	9 (23)	0
Decreased appetite	16 (26)	2 (3)	10 (25)	0
Pyrexia	15 (25)	0	3 (8)	0
Diarrhea	13 (21)	0	4 (10)	0
Hypertension	13 (21)	7 (11)	3 (8)	2 (5)
Malaise	13 (21)	0	6 (15)	0
Constipation	12 (20)	0	7 (18)	0
Epistaxis	12 (20)	0	3 (8)	0
Hypoalbuminemia	11 (18)	0	3 (8)	1 (3)
Proteinuria	11 (18)	0	3 (8)	0
Nausea	10 (16)	0	4 (10)	0
AESIs^a^				
Liver injury/liver failure	34 (56)	15 (25)	11 (28)	8 (20)
Ascites	20 (33)	2 (3)	1 (3)	1 (3)
Hepatic encephalopathy	3 (5)	3 (5)	0	0
Bleeding/hemorrhage events	21 (34)	3 (5)	6 (15)	1 (3)
Epistaxis	12 (20)	0	3 (8)	0
Hypertension	14 (23)	8 (13)	3 (8)	2 (5)
Proteinuria	12 (20)	0	3 (8)	0
GI hemorrhage events	6 (10)	3 (5)	2 (5)	1 (3)
Arterial thromboembolic events	1 (2)	0	0	0
Congestive heart failure	1 (2)	1 (2)	0	0
Pulmonary hemorrhage events	1 (2)	0	1 (3)	0
Venous thromboembolic events	1 (2)	0	0	0

*AESI* adverse event of special interest, *AFP* alpha-fetoprotein, *GI* gastrointestinal, *MedDRA* Medical Dictionary for Regulatory Activities, *TEAE* treatment-emergent adverse event

^a^AESI consolidated category term or MedDRA preferred term

## Discussion

The global REACH-2 study showed a significant survival benefit for ramucirumab in patients with HCC and AFP ≥ 400 ng/mL who had progressed on or were intolerant to sorafenib [20]. This prespecified subgroup analysis also showed a survival benefit for ramucirumab in the Japanese REACH-2 subpopulation. Ramucirumab was well-tolerated in the Japanese REACH-2 subpopulation, with an acceptable safety profile. Taken together, ramucirumab offers an active and well-tolerated second-line treatment option for Japanese patients with HCC and elevated AFP levels.

As observed in the overall REACH-2 study population [20], there were improvements in PFS, OS, and TTP for ramucirumab versus placebo in the Japanese REACH-2 subpopulation. Aside from the overall study results [20], this is the first report of an agent showing clinical benefit as second-line treatment in a population of Japanese patients with HCC following prior sorafenib. Additionally, the improvements in PFS and OS in the Japanese REACH-2 subpopulation were consistent with those observed for ramucirumab in the subpopulation of Japanese patients with AFP ≥ 400 ng/mL (*n* = 42) in the REACH study (PFS HR 0.261, 95% CI 0.391–0.986; OS HR 0.464, 95% CI 0.232–0.926) [21]. As death due to liver cirrhosis can confound the potential benefits of the investigational drug, PFS and TTP are suggested as surrogate endpoints in phase 3 trials for patients with advanced HCC treated with molecular targeted therapy, with a threshold of HR ≤ 0.6 for PFS and TTP being suggestive of improvement in OS [22, 23]. In the current analysis, the improvements in PFS and TTP (which reflect the antitumor effects of the investigational drug per se, whereas OS may be confounded by postdiscontinuation therapy) were marked, with an HR of 0.282 and 0.248, respectively. This finding is striking, given the characteristics of Japanese patients with advanced HCC described earlier, making it difficult to show positive results in the Japanese subpopulation consistent with those in the overall study population of a global study. A marked improvement in PFS (HR 0.214, 95% CI 0.089–0.519) has also been reported for regorafenib versus placebo in the Japanese subpopulation (*n* = 40) of the phase 3 RESORCE study, conducted in patients with HCC who had progressed on sorafenib; however, the improvement in OS (HR 0.901, 95% CI 0.391– ~ 20.80) was less marked [24].

Ramucirumab was well-tolerated in the Japanese REACH-2 subpopulation, with a safety profile consistent with that observed in the overall REACH-2 study population and the overall experience of ramucirumab monotherapy in HCC and gastric cancer [19, 21, 25, 26]. Most of the differences in TEAEs between the ramucirumab and placebo arms in the Japanese REACH-2 subpopulation were related to low-grade events, which were manageable. In particular, as observed in the overall REACH-2 study population, the increased incidence of the AESIs of liver injury/liver failure and bleeding/hemorrhage in the Japanese REACH-2 subpopulation was largely due to low-grade ascites and epistaxis, respectively. The incidence of ascites (24% vs. 18%) and hepatic encephalopathy (7% vs. 4%) was higher in ramucirumab-treated patients in the Japanese REACH-2 subpopulation than in the overall REACH-2 study population, which might be partly related to a longer duration of ramucirumab treatment (median duration 16 vs. 12 weeks). It is possible that the older age of the Japanese REACH-2 subpopulation compared with the overall REACH-2 study population (median age, ramucirumab/placebo: 71/68 vs. 64/64 years), and the higher rate of hepatitis C virus infection (etiology of HCC, ramucirumab/placebo 37%/44% vs. 24%/29%), likely accompanied by more advanced liver cirrhosis, might contribute to the higher incidence of ascites and hepatic encephalopathy in the Japanese REACH-2 subpopulation compared with the overall REACH-2 study population. However, the mechanism underlying this has not been established.

There were some differences between the Japanese REACH-2 subpopulation and the overall REACH-2 study population, including median duration of disease, which was longer in the Japanese REACH-2 subpopulation (ramucirumab: 43.70 months; placebo: 26.76 months) than in the overall REACH-2 study population (ramucirumab: 20.1 months; placebo: 17.6 months). Marked regional variations in the time from initial diagnosis to the start of sorafenib treatment, with longer times in patients in Japan, have been reported previously [13]. This is thought to be due to the intensive patient follow-up that occurs in Japan from the time the patient is first diagnosed with hepatitis or liver cirrhosis. In addition, prior locoregional therapy (81% vs. 64%) and TACE (71% vs. 53%) were more common in the Japanese REACH-2 subpopulation than in the overall REACH-2 study population; similar observations were made in the previous REACH clinical trial [21]. However, the median duration of sorafenib treatment was similar between the Japanese REACH-2 subpopulation (ramucirumab: 5.29 months; placebo: 5.32 months) and overall REACH-2 study population (ramucirumab: 4.1 months; placebo: 4.1 months), as was the percentage of patients who discontinued sorafenib because of progressive disease/intolerance in the Japanese REACH-2 subpopulation (ramucirumab: 88%/12%; placebo: 72%/28%) compared with the overall REACH-2 study population (ramucirumab: 84%/16%; placebo: 80%/20%). The use of postdiscontinuation systemic therapy was a little lower in the ramucirumab arm compared with the placebo arm (24% vs. 33%) in the Japanese REACH-2 subpopulation; however, the limited patient numbers makes it difficult to assess the effect of this difference in postdiscontinuation therapy on OS. In the overall REACH-2 study population, the use of postdiscontinuation systemic therapy was similar between the two treatment arms (27% vs. 28%), and sensitivity analysis showed that, after censoring for postdiscontinuation therapies, the difference in OS between treatment arms was clinically meaningful [20].

Pooling the patient-level efficacy data of the Japanese REACH-2 subpopulation and the Japanese REACH subpopulation with baseline AFP ≥ 400 ng/mL provided a larger patient population (*N* = 101); the pooled efficacy analyses also showed a survival benefit for ramucirumab in Japanese patients with HCC. The current subgroup analysis showed numerically longer survival of ramucirumab-treated patients in the Japanese REACH-2 subpopulation compared with the overall REACH-2 study population (median OS 10.2 vs. 8.5 months) [20]. Consistent with this, the current pooled analysis showed numerically longer survival of ramucirumab-treated patients in the pooled Japanese REACH-2/REACH subpopulations compared with the pooled overall REACH-2/REACH study populations (median OS 10.8 vs. 8.1 months) [27]. As discussed previously in relation to the REACH study [21], the reasons for numerically longer survival of ramucirumab-treated Japanese patients compared with the overall study population(s) require further elucidation, but may include differences in Japanese patient characteristics or disease management that result in greater benefit from an effective therapy [21]. The ramucirumab treatment benefit in the subgroup of patients in the pooled Japanese REACH-2/REACH subpopulations who were intolerant to sorafenib was consistent with that in the entire pooled Japanese REACH-2/REACH subpopulations; however, the small number of patients in this subgroup should be taken into account. Ramucirumab was well-tolerated in patients who were intolerant to sorafenib, with low rates of discontinuation due to treatment-related TEAEs.

Strengths of the analysis were the randomized, double-blind design of the REACH-2 study, the Japanese subgroup analyses being prespecified, and the pooled analysis with a similarly designed phase 3 study at the individual patient data level, to reinforce the results observed in the Japanese REACH-2 subpopulation by increasing the number of Japanese patients in the efficacy analyses. The post hoc nature of the pooled efficacy analyses was a limitation of the analysis.

In conclusion, this subgroup analysis of the Japanese patients enrolled in the REACH-2 study showed a clinically meaningful benefit for ramucirumab, accompanied by a manageable safety profile, in Japanese patients with HCC who had previously received sorafenib and had baseline AFP ≥ 400 ng/mL. The clinical benefits of ramucirumab observed in the Japanese REACH-2 subpopulation were in line with those observed for the overall REACH-2 study population. Thus, ramucirumab represents a promising treatment option in the second-line setting, in particular, in Japanese patients with advanced HCC and elevated AFP. Further analysis to explore the characteristics of the Japanese REACH-2 subpopulation is planned.

## Electronic supplementary material

Below is the link to the electronic supplementary material.

Supplementary file1 (PDF 133 kb)
