# Supplementary material for: Ramucirumab after prior sorafenib in patients with advanced hepatocellular carcinoma and elevated alpha-fetoprotein: Japanese subgroup analysis of the REACH-2 trial
Source: J Gastroenterol. 2020 Feb 27;55(6):627–39. doi: 10.1007/s00535-020-01668-w (PMC7242235; doi:10.1007/s00535-020-01668-w)
Supplement: Supplementary file 1 — Supplementary file1 (PDF 133 kb) [file 535_2020_1668_MOESM1_ESM.pdf]

Title: Ramucirumab after prior sorafenib in patients with advanced hepatocellular carcinoma and elevated alpha-fetoprotein: Japanese subgroup analysis of the REACH-2 trial

Journal: *Journal of Gastroenterology*

Authors: Masatoshi Kudo, Takuji Okusaka, Kenta Motomura, Izumi Ohno, Manabu Morimoto, Satoru Seo, Yoshiyuki Wada, Shinpei Sato, Tatsuya Yamashita, Masayuki Furukawa, Takeshi Aramaki, Seijin Nadano, Kazuyoshi Ohkawa, Hirofumi Fujii, Toshihiro Kudo, Junji Furuse, Hiroki Takai, Gosuke Homma, Reigetsu Yoshikawa, Andrew Zhu

Corresponding author: Masatoshi Kudo; Department of Gastroenterology and Hepatology, Kindai University Faculty of Medicine, 377-2 Ohno-Higashi, Osaka-Sayama, Osaka 589-8511, Japan; m-kudo@med.kinki.ac.jp

Supplementary Table 1 Postdiscontinuation systemic therapies in the Japanese REACH-2 subpopulation

Systemic therapy<br>n (%)	Ramucirumab<br>(n = 41)	Placebo<br>(n = 18)
Overall	10 (24)	6 (33)
Chemotherapy	4 (10)	2 (11)
Cisplatin	2 (5)	0
Fluorouracil	2 (5)	0
Gimeracil w/oteracil potassium/tegafur	3 (7)	1 (6)
Tegafur/uracil	1 (2)	1 (6)
Immunotherapy/immunomodulatory agents	4 (10)	1 (6)
Atezolizumab	1 (2)	1 (6)
Ipilimumab	2 (5)	0
Lirilumab	1 (2)	0
Mogamulizumab	1 (2)	0
Nivolumab	3 (7)	0
Pegylated interferon alpha-2A	1 (2)	0
Targeted small molecule inhibitors	6 (15)	3 (17)
Regorafenib	4 (10)	2 (11)
Sorafenib	4 (10)	2 (11)
Targeted antibody	1 (2)	1 (6)
Codrituzumab	1 (2)	1 (6)
